# Treatment of Fracture of the Lower Jaw by Interdental Splints

**Published:** 1868-08

**Authors:** Thomas Brian Gunning


### ARTICLE II.

### *Treatment of Fracture of the Lower Jaw by Interdental Splints.*

By Thomas Brian Gunning.

(Continued.)

February 16th. The general condition of the soft parts much improved, but no difference in the right articulation. It is yet impossible to set the halves of the jaw together properly, without bringing the left half down to meet the other. The right condyle, although apparently in its place in the glenoid fossa, is not so, as the back teeth on this side meet too soon, so that the teeth cannot close, at the canines, by about two lines. The *left half* was therefore brought forward at the condyle, *until a full quarter of an inch down at*

*the wisdom tooth and the same at the canine.* In this position it was set up in the wax resting upon the upper teeth and the bite taken. When the casts were placed in the wax bite to form the mould for the splint, the upper and lower right wisdom teeth were separated about a line. This was done in the hope that when the splint was applied the parts might yield, so as to allow the condyle to fall away some.

February 19th. On applying the splint the right fragment would not go up into place, even under much pressure, until the parts between the crowns of the wisdom teeth was all cut away—showing that no improvement had taken place in the joint to this time. The right wisdom tooth is hard against the one above, while the canine teeth and the other wisdom tooth are considerably below the upper ones. Splint left unfastended.

February 20th. Patient very comfortable, except that the edge of the splint cuts the gum a little. The splint was removed and made easy, then screwed to the first right upper molar and left canine, and to both lower canines. The jaws are held as close together as the back teeth permit, for as all the four upper incisor teeth have been absent for some time, the opening in the splint is large enough without depressing the lower jaw. A channel is cut in each side of the splint, that the saliva from the parotid glands may get into the mouth.

February 24th. Patient very comfortable and much pleased with the splint. All going on well.

March 16th. Swelling set in over right condyle and ramus in the beginning of the month, but passed off. About the same time the part under the symphysis opened, but closed up after a teaspoonful of pus had discharged. The swelling on the neck of the right condyle is still very painful; doing well in other respects.

March 30th. Swelling all gone, except the small lump near right condyle, which is still painful. Left central incisor (next to the fracture) quite tender, and pus discharging from its socket.

April 12th. Tooth better. Swelling near condyle less painful.

April 22d. Swelling near right condyle much less painful. Splint has been worn sixty-two days, and been on without a moment's intermission just sixty-one days. Removed it and good union found. Upper part of the splint cut off, and the *jaw allowed to move*—the lower part being put on again, as the union is not yet stiff enough. The jaw is therefore left in a splint, like Fig. 1, but still screwed to the canine teeth.

May 18th. Splint dispensed with. Jaw firmly united and the same shape as before the accident. *Also going into its place as regards the upper jaw*, the top of the splint, where the points of the upper teeth rest, having been cut down about once a week since the jaw has been allowed to move. It continued to improve and go up closer to the upper teeth until the beginning of July when it was nearly in place. The jaw moved very well *up* and *down*, but the right condyle had very little ability to come forward in the lateral movement of the jaw.

September 8th. The patient is out of town; but I have heard from several sources that the jaw is all right.

September 15th. The patient's father says the lateral motion is nearly perfect again, and the jaw in place.

This case shows the necessity of some intervening support between the teeth in some cases during treatment, and therefore affords another argument in favor of interdental splints.

Case 7.—P. N., thirty-six years old, was struck with a club on the left side of the jaw, August 19, 1866. Went to the Demilt Dispensary, from whence he was brought to me, August 22. Find the jaw-bone broken on both sides. The lower lip and parts covering the mental process have little sensation, owing to the separation of the inferior dental nerves. Fracture on the left side, between the biscupids; it is square across, vertical and smooth. The biscupid teeth quite firm. The first downward and forward about four lines from the second. The other fracture is through the socket of the lower right wisdom tooth, leaving one root in front fragment, while the crown of the tooth is held by its back root, in the part attached to the ramus. Fracture passes down, inclining to the angle. The back fragment keeps forward and up, so that the wisdom tooth strikes against the upper teeth, while the forward fragment is full half an inch down when at rest. Much swelling, pain and discharge of pus. The jaw settles over to the right. The teeth above and below are all present, except the lower left wisdom tooth, and pretty firm, except the one in the fractured socket. Both upper and lower teeth show distinctly where their antagonists closed against them. The lower jaw shuts a trifle outside the upper at the *right* bicupids, owing to a very peculiar curl outward of the *left* angle, which has caused the muscles to swing the jaw over somewhat. the patient says this irregularity was caused by the kick of a mule, when he was about nine years old. Tied the bicuspids together with silk, and took a wax impression of the fourteen teeth, leaving out the elevated wisdom tooth, of which an impression was taken separately. The parts were not precisely placed, therefore the plaster-cast was sawed apart between the bicuspids, and adjusted by the cast of the upper jaw. The wisdom tooth was added in the same way. The lower cast then included the fifteen teeth of the lower jaw, *all in place.* It was placed against the upper cast, and *both set in an articulator.* The jaws were then opened nearly three-eighths of an inch, and a gutta-percha splint made. This was tried in the mouth, and being right was trimmed to the form required for the splint; then, with the upper and lower casts,set in a vulcanizing flask, with the female screws all in place. After the plastər had set the flask was made quite warm, in order that the plaster teeth should not be broken when drawing out the gutta-percha to make room for the rubber. The opening in front of the splint reached from one canine to the other, and from the points of the upper teeth to those below, and holes were made through the sides for the saliva.

August 25th. When applying the splint considerable difficulty was experienced in getting the jaw into place owing to the pain and displacement of the fragments; but after placing packthread around the front teeth, and pulling the jaw over to the left, every part went up into the splint, although scarcely as high as they should, the splint being rather tight, because of the improper omission of the two or three coats of silicious varnish usually given to the plaster teeth before packing the soft rubber. The splint was screwed to both canines, on the left side, and to the upper first molar and lower first bicuspid on the right.

August 27th. Swelling and pain lessened very much.

August 28th. All going well; patient wants to know if he may go to work.

August 29th. Parts begin to look natural. Patient sleeps well, except when coughing, through a severe cold.

August 31st. Patient quite comfortable, except when coughing at night. Has begun to work at his trade, (glass cutting).

September 5th. Patient has been out in the country to see a sick relative. Removed the splint and cut away some parts pressing too hard on the roof of the mouth. Fractures making fair progress. Took away the right wisdom tooth, it being very loose, as in addition to the loss of one root the tooth had been further loosened by attempts to extract it before the patient went to the dispensary. Replaced the splint.

September 8th. Sensation returning to lower lip, etc. Doing well in every particular. The right back fragment has no tooth to hold it, but the muscles keep it firm against the portion in front.

September 15th. All going on well.

Sept. 18th. Pus again discharging profusely from the right fracture. Patient says, the bone moves; he points to the coronoid process. When the temporal and masseter muscles are brought into action, crepitus can be distinctly felt, especially if the finger be placed on the left angle where there is no swelling.

Sept. 20th. Displacement of the right ramus outward, forward and upward.

Sept. 21st. Swelling and pain increased since yesterday. On removing the splint, I find good but flexible union on the left side; the right fracture proves to be very oblique and diagonal to the thickness of the bone; it commences outside the second molar, passes through the socket of the third (the extracted tooth,) and terminates somewhere on the inside, short of the angle. Since Sept. 5th, when the wisdom tooth was extracted, this fragment has had nothing to hold it back in place, except the roughness of the fractured surfaces, which may have given way under the action of the unusually strong muscles and the jarring of a severe cough. When describing the splints before the Academy of Medicine, I suggested that when necessary, metallic points could be arranged in them to go into the bone. I now decided to apply one in this case, for as the line of fracture averages three inches around the bone, a salient, edge one inch and a half wide on each fragment, is pressing into the periosteum and other tissues. This can all be remedied by the aid of a piece of wire, which may go into the muscles, etc., perhaps a quarter of an inch, and press against the bone even less than that. A steel hook was, therefore, screwed into the end of the splint, just below the back corner of the upper wisdom tooth. The wire is a line in diameter and three quarters of an inch long, clear of the splint. It is bent, so as to go down outside the bone where the ramus starts from the body. The point of the hook goes through the buccinator muscle and rests firmly on the bone. Firm pressure on the splint forced the ramus back, and the splint went on to the upper teeth, but at the expense of carrying the front of the lower jaw too much to the right, as the overlapping of the fragments did not yield readily. Packthread was passed around the right bicuspid and canine, and after drawing the front of the jaw to the left for ten or twelve minutes, the fragments came into position and the teeth went up in the splint. The bone was then quite firm, the action of the muscles causing no motion in it whatever.

Pain was felt for several days near the condyle and in the front of the ear, with occasional stinging in the temple ; but this, with the swelling and suffering of the previous displacement, rapidly passed away.

Oct. 4th. All the parts are looking better than at any time since the injury. The pus is much diminished, and the bone is held quite still.

Oct. 8th. Only a little pus to be seen, but a piece of the alveolar which lies in the gum on the outside of the right second molar is nearly detached. No motion has been felt in the ramus since the hook was applied,

Oct. 11th. Removed the loose piece of alveolar easily.

Oct. 19th. Patient failed to call on the 15th and 18th, but came to day, quite drunk. The splint is firm, however, and the bone doing well.

Oct. 26th. His wife called and said he had been in jail since the evening of the 19th, and that for a week previous he had been drunk nearly all the time. She was afraid his jaw was injured, as he had thrown himself about very much and vomited frequently.

Oct. 28th. I called at the prison. He looks thin and pale, but the jaw is doing well, and the splint secure.

Nov. 24th. He has been out some time; I removed the splint to-day. The left side of the jaw is quite strong and there is good but flexible union on the right side. The hook has been worn sixty-four days without a moment's intermission ; the hole left in the gum is just the size of the wire and the parts around are quite healthy. Splint dispensed with.

Dec. 10th. The callus has stiffened very much since the splint was left off, and both sides of the jaw are now used in eating.

The man was spared much pain by adjusting the casts by the articulator. In fact, it would have been hardly possible to set the fragments of the bone in place and hold them there while taking the impressions and bite, the case being so extremely severe in every particular. All attempts to hold it in place by bandage, even temporarily, were ineffectual.

In this case neither weight nor distension could have displaced the bone, for the ramus was drawn upwards and the swelling had subsided. The temporal and associate muscles must therefore have been the only cause of the displacement, although opposed by the body of the jaw, which was held still by the splint. This case, therefore, with the others used to illustrate the treatment, shows that the muscles are active causes of displacement, as distinctly intimated by me throughout the subject, and formally stated in the paper read before the New York Academy of Medicine, June 1, 1864.

Other cases treated and seen by me also demonstrate that the opinion expressed so decidedly by Malgaigne and entertained bv Hamilton, as to the effects of the impulse given by the cause of fracture upon displacement, is erroneous. For the impulse being exhausted in deciding the position, direction, and extent of the injury the bone and surrounding tissues, the bone is then *surrendered* to the muscles which affected it before and at the time of fracture, and still continue to do so, according to *the condition in which it and they are left.*

(*To be Continued.*)

---

